# Comparison between EQ-5D and SF-6D Utility in Rural Residents of Jiangsu Province, China

**DOI:** 10.1371/journal.pone.0041550

**Published:** 2012-07-27

**Authors:** Hui Jin, Bei Wang, Qian Gao, Jianqian Chao, Siyuan Wang, Lin Tian, Pei Liu

**Affiliations:** 1 Department of Epidemiology and Health Statistics, Southeast University, Nanjing, China; 2 Key Laboratory of Environmental Medicine Engineering, Ministry of Education, School of Public Health, Southeast University, Nanjing, China; 3 Department of Medical Insurance, Southeast University, Nanjing, China; The University of Hong Kong, Hong Kong

## Abstract

**Background:**

The SF-6D and EQ-5D are widely used generic index measures as health-related quality of life. We assessed within-subject agreement between SF-6D and EQ-5D utilities with different preference weights, and their validities in measuring Chinese rural residents, before and after standardization scores.

**Methodology/Principal Findings:**

Rural residents over 18 years old were interviewed using EQ-5D and SF-6D in Jiangsu Province, China. EQ-5D utility-scoring algorithms were used from three conversion tables from the United Kingdom, Japan, and the United States. Validities, Sensitivity and agreement between instruments were computed and compared. Factors affecting utility difference were explored with multiple liner regression models. Scores with standardization intervals of 0–1 in the two instruments were analyzed by the use of the above methods again. In 929 respondents, relative efficiency statistic and receiver operating characteristic curves analysis showed SF-6D to be the more efficient, followed by the EQ-5D model in Japan weights. Bland–Altman plot analysis showed paired SF-6D/EQ-5D in UK weights had better agreement. Though some risk factors were found, multiple liner regression demonstrated most coefficients were weaker than 0.2, and all R^2^ values were less than 0.06. Standardization did not significantly influence these results except scores' value.

**Conclusions/Significance:**

SF-6D and next EQ-5D in Japan weights could be used for Chinese rural residents. Further research with larger sample size of population is needed to establish and determine the feasibility of standardization score.

## Introduction

In light of the fact that evaluating health related-quality of life (HRQoL) currently operates without a gold standard, it is important to understand the real state of health by comparing different instruments. Some studies have focused on comparisons between European quality of life (EQ-5D) and Short Form of the Medical Outcomes Study Questionnaire (SF-6D) in measuring discrepancies from the general population [1], [2] and patients [3], [4], [5], [6], [7].

In EQ-5D, the best-known preference weights were derived from samples in a UK population, which may be applied to other populations when country-specific weights are not available, such as in China [8]. Now different weights in EQ-5D were randomly used in China and other countries, which cause the occurrence of different scores for the same population. Therefore, it is necessary to ensure the suitable weight in current ones for Chinese population. Moreover, using the same preference weights, some evidence has suggested that valuations of health states could differ for people in different countries owing to differences in demographic backgrounds [9], [10], including self-reported score. Also, it is worth noting the potential complexity on the comparison from different intervals of utility score between EQ-5D (−0.59 to 1.00 or −0.11 to 1.00) and SF-6D (0.32 to 1.00). However, both of them were used to evaluate the real health related-quality of life and compared with each other in many studies despite the different scales. It is difficult to be understood that life of quality was negative value, or the same patient had significant distinct in utility scores. Therefore, it is of great significance to standardize these intervals (0 to 1) for the understanding and comparisons. Have the standardization similar effects as the non-standardization in these instruments? Furthermore, more studies have been carried out on patients than on the general population, on urban rather than on rural residents in China [11], [12] and others countries [5], [6], [7], so that the applicability of the above instruments was not taken into account for the latter.

Therefore, this study provides an opportunity to examine Chinese rural residents' HRQoL as measured by EQ-5D with three countries' preference-weighted scores and SF-6D instruments to test the validity and sensitivity of these instruments and assess within-subject agreement between them before and after standardization scores ranging from 0 to 1.

## Materials and Methods

### Study Subjects

The target population for the study was Jiangsu's rural residents, aged 18 and older, with rural hukou. A multistage, stratified, random sampling procedure was employed, aiming at generating a sample representing the age, sex, and socioeconomic status distribution in the target population. Due to limited resources, the target sample size was restricted to 1,000 individuals. The subjects were sampled from three counties (Taixing, Danyang, and Zhangjiagang) in Jiangsu Province, China, in 2010. The subjects were assigned to 13 regions according to population size. And 25 to 30 households were randomly selected for interview in these regions. Temporary residents were excluded. Following informed consent, each subject was interviewed by a trained interviewer using a standardized questionnaire containing the sociodemographic information, the medical conditions, the EQ-5D/visual analog scale (VAS) and SF-6D. This study was approved by the Ethics Committee of the Jiangsu Provincial Center for Disease Control and Prevention. We obtained written informed consent from all participants involved in our study. The data were analyzed anonymously.

### Utility Instruments

The SF-6D algorithm is described in detail elsewhere [13]. The SF-6D utility-scoring algorithm was derived from a representative sample of the UK general population with Standard Gamble (SG) method, ranging from 0.32 to 1.00. The Hong Kong Chinese version and HK scoring algorithm of SF-6D was adopted [14]. To assess differences in the EQ-5D algorithm, scores were compared from three conversion tables of the United Kingdom (EQ-5D-UK) [8], Japan (EQ-5D-JP) [15], and the United States (EQ-5D-USA) [16], using time tradeoff (TTO)-based preference scores. The scores ranged from −0.59 to 1.00 in the United Kingdom weights and from −0.11 to 1.00 in Japan and the United States. The EQ-5D Visual Analogue Scale (VAS) records the respondent's self-rated health status on a VAS. The simplified Chinese version of EQ-5D/VAS in this study is an official version authorized by the EuroQol Group.

### Statistical Analysis

Continuous variables are presented as mean standard error (SE), while categorical variables are shown as a proportion of the sample. EQ-VAS scores were divided by 100 to generate values between 0 and 1.

Convergent validity of the EQ-5D and SF-6D was assessed by examining their association with EQ-VAS classified by different cutoff values [17]. The validity coefficient was computed as Spearman's rank correlation coefficient [12]. The efficiency of EQ-5D and SF-6D to detect the relevant differences was compared using relative efficiency (RE) statistic and receiver operating characteristic (ROC) curves. The area under the ROC curves (AUC) was computed to compare the discriminative properties of these instruments (AUC≥0.5).

Agreement among these instruments was assessed by means of Bland-Altman plots [18], the limit of agreement (LOA) being greater than 0.95. To determine whether the subjects' socioeconomic status was related to the utility difference between EQ-5D and SF-6D, multiple linear regression (MLR) was used in all entry models. The standard adjustments were as follows: SF-6D value minus 0.32 and then divided by 0.68, EQ-5D-UK value added 0.59 and then divided by 1.59, EQ-5D-JP/EQ-5D-USA value added 0.11 and then divided by 1.11. After the corresponding adjustment was done to obtain identical intervals between SF-6D and EQ-5D for 0–1, the standard results from the above analysis methods were compared with the previous nonstandard ones.

All statistical analyses based on complex sampling data were conducted using SAS version 9.1 with the programmes, such as surveyfreq, surveymeans and surveyreg (SAS Institute Inc., Cary, NC, USA.).

## Results

There were 929 (the response rate 92.9%) SF-6D and EQ-5D forms evaluated in our study, with no missing items eligible for analysis, while 71 subjects were excluded for refusal to answer questions or urban residents. The sample sociodemographic characteristics were shown in Table 1 and 2. The scores' value increased in EQ-5Ds and decreased in SF-6D after the standardization of the interval.

**Table 1:** Variable definition and respondents' characteristics of EQ-5D and SF-6D utility scores (n = 929).

Variables		n (%)	Variables		n (%)
Gender	Male	496(53.4)	Age(years old)		39.28(13.25)*
	Female	433(46.6)	Annual family income		7267.93(10113.36)*
Marriage	Married	753(81.1)	Education	Lower ≤6 years	118(12.7)
	Unmarried	158(17.0)		Middle 7–12 years	485(52.2)
	Divorced	18(1.9)		Higher >12 years	326(35.1)
Family	1 person	11(1.2)	Health	Full coverage	34(3.7)
size	2–4 persons	530(57.0)	insurance	Partial coverage	825(88.8)
	>4 persons	388(41.8)	coverage	No insurance	70(7.5)
Chronic	Yes	110(11.8)	Infectious	Yes	16(1.7)
conditions	No	819(88.2)	conditions	No	913(98.3)

* Mean (Standard deviation). EQ-5D, EuroQol; SF-6D, Short Form 6D. EQ-5D-UK, EQ-5D in UK weights; EQ-5D-JP, EQ-5D in Japan weights; EQ-5D-USA, EQ-5D in USA weights; Chronic conditions, Has diagnosed chronic conditions in last 6 months. The currency exchange rate is 1 US $ to 6.4 Yuan.

**Table 2:** Variable definition and respondents' characteristics of EQ-5D and SF-6D utility scores (n = 929).

Variables		Mean (SD)	Min, Max	CE* n(%)
Non-standardization	SF-6D&	0.893 (0.099)	0.380,1.000	220(23.7)
scores	EQ-5D-UK	0.904 (0.162)	−0.350,1.000	582(62.6)
	EQ-5D-JP	0.899 (0.139)	0.190,1.000	582(62.6)
	EQ-5D-USA	0.922 (0.121)	0.080,1.000	582(62.6)
	VAS	0.863 (0.117)	0.100,1.000	101(10.9)
Standardization	SF-6D&	0.842(0.146)	0.085,1.000	220(23.7)
scores	EQ-5D-UK	0.939(0.102)	0.152,1.000	582(62.6)
	EQ-5D-JP	0.909(0.125)	0.270,1.000	582(62.6)
	EQ-5D-USA	0.930(0.109)	0.174,1.000	582(62.6)
	VAS	0.863 (0.117)	0.100,1.000	101(10.9)

* CE, ceiling effect. n, the number with full score. EQ-5D, EuroQol; SF-6D, Short Form 6D. EQ-5D-UK, EQ-5D in UK weights; EQ-5D-JP, EQ-5D in Japan weights; EQ-5D-USA, EQ-5D in USA weights.

A strong ceiling effect was observed (Table 2): the highest percentage of the ceiling effect appeared with mobility, self-care, and usual activities in EQ-5D, and role limitation in SF-6D (Table 3 and 4). For rural residents, the mental and vitality dimensions were associated with more serious problems in SF-6D, while pain/discomfort and anxiety/depression were seen in EQ-5D.

**Table 3:** Distribution of each EQ-5D or SF-6D dimension (n = 929).

EQ-5D	Degrees (%)			
Dimensions	None	Moderate	Extreme	ME*
Mobility	895(96.3)	32(3.4)	2(0.2)	34(3.6)
Self-care	914(98.4)	9(1.0)	6(0.6)	15(1.6)
Usual Activities	901(97.0)	24(2.6)	4(0.4)	28(3.0)
Pain/Discomfort	683(73.5)	238(25.6)	8(0.9)	246(26.5)
Anxiety/Depression	713(76.7)	205(22.1)	11(1.2)	216(23.3)

* ME, moderate or extreme. EQ-5D, EuroQol.

**Table 4:** Distribution of each EQ-5D or SF-6D dimension (n = 929).

SF-6D*	Degrees (%)						
Dimensions	1	2	3	4	5	6	2–6
Physical functioning	611(65.8)	241(25.9)	52(5.6)	18(1.9)	3(0.3)	4(0.4)	318(34.2)
Role limitation	819(88.2)	69(7.4)	25(2.7)	16(1.7)	-	-	110(11.8)
Social functioning	586(63.1)	272(29.3)	58(6.2)	12(1.3)	1(0.1)	-	343(36.9)
Pain	584(62.9)	203(21.9)	106(11.4)	26(2.8)	5(0.5)	5(0.5)	345(37.1)
Mental health	432(46.5)	370(39.8)	106(11.4)	13(1.4)	8(0.9)	-	497(53.5)
Vitality	346(37.2)	475(51.1)	61(6.6)	28(3.0)	19(2.1)	-	583(62.8)

* SF-6D, Short Form 6D.

### Validation Sensitivity of EQ-5D and SF-6D

Convergent validity was demonstrated by moderate correlation coefficients (*r*≥0.349) between EQ-5D/SF-6D and VAS, strong (*r*≥0.574) between SF-6D and EQ-5D, and very strong (*r*≥0.999) between different EQ-5Ds (Table 5). A significant difference in utility scores was observed among different levels of VAS for these instruments (P<0.0001). The RE statistic calculation showed that EQ-5D-JP had a greater efficiency at detecting a difference in VAS scores under its different cutoff values than EQ-5D-UK and EQ-5D-USA; however, SF-6D's RE was higher than EQ-5D-JP's except for the VAS cutoff between 0.80–0.90 (Table S1). The orders of the AUC scores were as follows: SF-6D>EQ-5D-JP>EQ-5D-UK or EQ-5D-USA. The results after standardization scores had a similar effect on the sensitivity except the mean scores.

**Table 5:** Correlation matrix for EQ-5D, Short-Form 6D and VAS.

Table	EQ-5D-UK	EQ-5D-JP	EQ-5D-USA	SF-6D	VAS
EQ-5D-UK	1.000				0.350*
EQ-5D-JP	0.999*	1.000			0.351*
EQ-5D-USA	1.000*	0.999*	1.000		0.349*
SF-6D	0.574*	0.575*	0.574*	1.000	0.417*

* All correlations are significant at the 0.01 level (2-tailed). The validity coefficient is computed as Spearman's rank correlation coefficient. EQ-5D, EuroQol; EQ-VAS, EuroQol Visual Analog Scale; SF-6D, Short Form 6D. EQ-5D-UK, EQ-5D in UK weights; EQ-5D-JP, EQ-5D in Japan weights; EQ-5D-USA, EQ-5D in USA weights.

### Evaluation of Agreement

**Figure 1:**
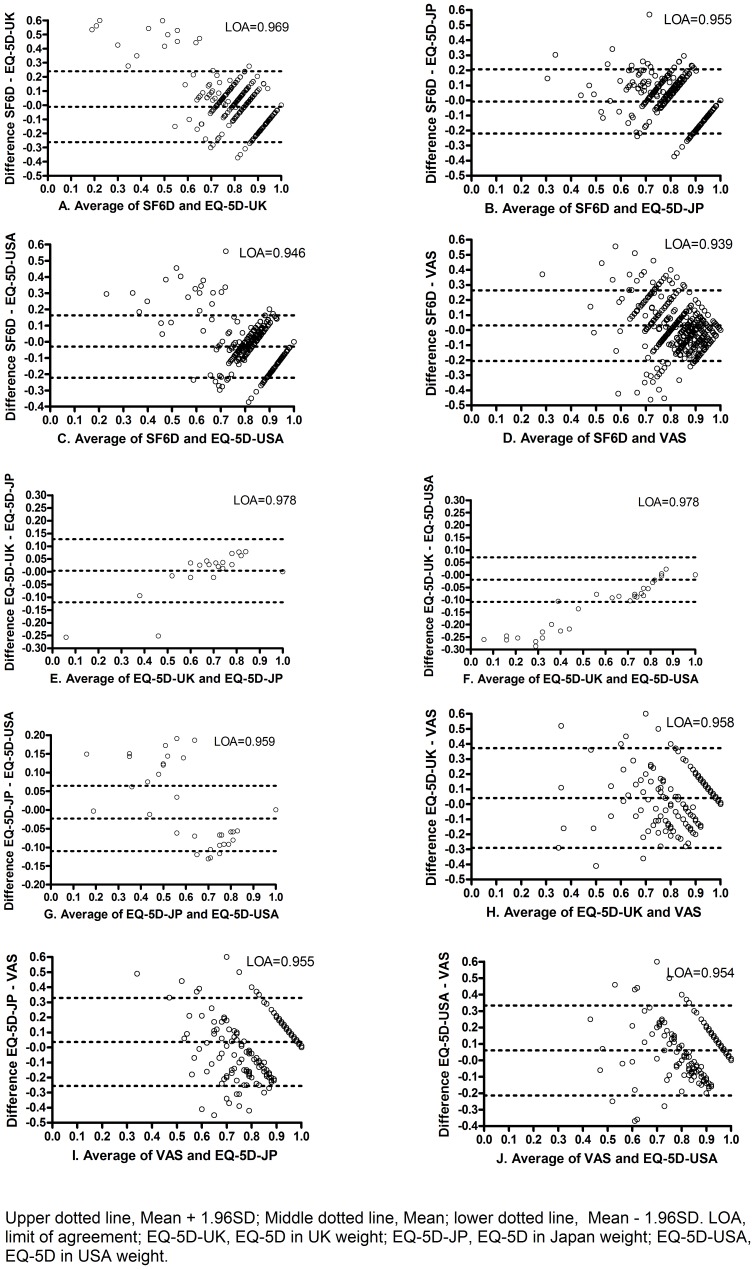
Difference against mean between SF-6D and EQ-5D.

In the non-standardization model, SF-6D showed better agreement with EQ-5Ds than with VAS; EQ-5D-UK and EQ-5D-JP/EQ-5D-USA had the highest LOA of 97.8%, while EQ-5D-JP and EQ-5D-USA had the lower LOA of 95.9%; different EQ-5D had good agreement with VAS (LOA>0.95) (Figure 1). Similar results were found in the standardization model.

### Factors Affecting Utility Difference between EQ-5D and SF-6D

Noticeably, when SF-6D or VAS was compared with different EQ-5Ds, middle education demonstrated lower scores difference between SF-6D and EQ-5D than higher education, whether adjusting scores ranging from 0 to 1 (Table S2). Other factors such as age, marriage and acute medical condition influenced their difference. Full or partial coverage showed less difference changes in utility scores than self-expense among EQ-5Ds and VAS. After standardization, most of these variables demonstrated similar association for the EQ-5Ds. However, these coefficients had a weak strength of less than 0.2, and all R^2^ values were less than 0.05.

## Discussion

In this study, we provide evidence of the validity and sensitivity of EQ-5D with different preference weights and SF-6D in general Chinese rural residents. However, some qualifications have to be made.

First, for distinguishing self-reported health status, RE and ROC analysis showed SF-6D to be the more efficient [6], followed by the EQ-5D model, in Japan. SF-6D includes broader aspects of HRQoL, such as role and social functioning, and has a greater response level for each domain [19]. This can make the description of health status more comprehensive, and patients would be more likely to find the best description for their status. In fact, the five-level version of EQ-5D is under development [20]. Also, it is one of the reasons why EQ-5D utility scores tend to be higher than SF-6D scores in healthier population [5], [12]. The Japan scheme provided better convergent and known-groups validity than the UK and US schemes did in this sample. These results may reflect the fact that China is an Asian country, whose culture is closer to Japan than to the United Kingdom and the United States. Noticeably, SF-6D's RE was higher than EQ-5D-JP's except for the VAS cutoff between 0.80–0.90. The phenomenon was related to selection of VAS, which was self-reported scores and underestimated by Chinese rural residents; moreover, the interval of 0.80–0.90 included ones from healthy people with conservative self-evaluation. Moreover, being different from other studies [12], MLR analysis implied the ability of understanding [12], influenced by the education levels, and could potentially introduce systematic bias resulting from possible differences in rural residents' experience. It is necessary to further follow up more rural residents and give more reasonable evaluation, especially for healthy people.

Second, EQ-5D had a greater stronger ceiling effect than SF-6D, and this may limit its ability to discriminate within the general population with mild to moderate symptoms. The relatively small sample size of chronic patients with mild symptoms might aggregate the high ceiling effect observed. Similar phenomena have been found in chronic prostatitis patients in China [12]. Several statistical methods have been proposed to address ceiling effects, such as Tobit models, the censored least absolute deviation approach, two-part models(TPM) and latent class models (LCM), which were compared by Huang et al [21]. Huang et al suggested the LCM and TPM with a log-transformed were superior to other approaches.

Third, Standardization of scores could be introduced into the direct comparison between the two instruments. The idea of standardization scores is based on an assumption that the scores from different instruments could be conveniently compared and be easily understood by readers at the same interval, ignoring various preferences methods and models. The standardization scores for different measurements had similar effects to nonstandard scores except the scores' value in the study. The value in SF-6D decreased while the ones in EQ-5Ds increased slightly, potentially owing to different dimensions and higher proportion in healthy people. The phenomenon would be weakened when the standardization of the interval was used in patients' evaluation of life of quality in these instruments. However, the standardization scores were not applicable in the instruments with non-linear scale, and they maybe conceal the truth of people health. Further research with larger sample size of population, especially for patients with clear definition, is needed to establish and determine the feasibility of standardization score.

## Supporting Information

Table S1 **Efficiency of EQ-5D and SF-6D to detect relevant difference.**
(DOC)

Table S2 **Multiple linear regression analyses for utility difference between EQ-5Ds and SF-6D.**
(DOC)
