# Supplementary material for: Comparison between EQ-5D and SF-6D Utility in Rural Residents of Jiangsu Province, China
Source: PLoS One. 2012 Jul 27;7(7):e41550. doi: 10.1371/journal.pone.0041550 (PMC3407238; doi:10.1371/journal.pone.0041550)
Supplement: Table S1 — Efficiency of EQ-5D and SF-6D to detect relevant difference. (DOC) [file pone.0041550.s001.doc]

Table S1. Efficiency of EQ-5D and SF-6D to detect relevant difference

|  |  |  |  | t test | |  | ROC curve | |
| --- | --- | --- | --- | --- | --- | --- | --- | --- |
| Measure | VAS | n | Mean(SE) | t statistic | P-value | RE† | AUC | 95%CI |
| SF-6D | ＞0.75 | 799 | 0.900(0.004) | 5.15 | 0.001 | 1.594 | 0.736* | 0.690,0.782 |
|  | ≤0.75 | 130 | 0.828(0.013) |  |  |  |  |  |
| EQ-5D-UK | ＞0.75 | 799 | 0.915(0.009) | 3.30 | 0.008 | 1.022 | 0.684* | 0.631,0.736 |
|  | ≤0.75 | 130 | 0.817(0.022) |  |  |  |  |  |
| EQ-5D-JP | ＞0.75 | 799 | 0.913(0.009) | 3.79 | 0.004 | 1.173 | 0.684* | 0.632,0.736 |
|  | ≤0.75 | 130 | 0.829(0.014) |  |  |  |  |  |
| EQ-5D-USA | ＞0.75 | 799 | 0. 932(0.007) | 3.23 | 0.009 | 1 | 0.684* | 0.631,0.736 |
|  | ≤0.75 | 130 | 0.857(0.017) |  |  |  |  |  |
| SF-6D | ＞0.80 | 632 | 0.910(0.005) | 6.28 | 0.001 | 0.924 | 0.712* | 0.677,0.748 |
|  | ≤0.80 | 297 | 0.849(0.007) |  |  |  |  |  |
| EQ-5D-UK | ＞0.80 | 632 | 0.931(0.008) | 6.30 | 0.000 | 0.926 | 0.680* | 0.642,0.719 |
|  | ≤0.80 | 297 | 0.840(0.010) |  |  |  |  |  |
| EQ-5D-JP | ＞0.80 | 632 | 0.929(0.006) | 8.61 | 0.000 | 1.266 | 0.681* | 0.642,0.719 |
|  | ≤0.80 | 297 | 0.845(0.010) |  |  |  |  |  |
| EQ-5D-USA | ＞0.80 | 632 | 0.943(0.005) | 6.80 | 0.001 | 1 | 0.680* | 0.641,0.718 |
|  | ≤0.80 | 297 | 0.875(0.007) |  |  |  |  |  |
| SF-6D | ＞0.85 | 574 | 0.910(0.006) | 5.60 | 0.000 | 0.712 | 0.703* | 0.669,0.737 |
|  | ≤0.85 | 355 | 0.858(0.007) |  |  |  |  |  |
| EQ-5D-UK | ＞0.85 | 574 | 0.930(0.008) | 7.67 | 0.000 | 0.975 | 0.658* | 0.621,0.695 |
|  | ≤0.85 | 355 | 0.858(0.007) |  |  |  |  |  |
| EQ-5D-JP | ＞0.85 | 574 | 0.929(0.007) | 9.04 | 0.000 | 1.149 | 0.659* | 0.622,0.696 |
|  | ≤0.85 | 355 | 0.860(0.009) |  |  |  |  |  |
| EQ-5D-USA | ＞0.85 | 574 | 0.943(0.006) | 7.87 | 0.000 | 1 | 0.658* | 0.621,0.695 |
|  | ≤0.85 | 355 | 0.888(0.005) |  |  |  |  |  |
| SF-6D | ＞0.90 | 302 | 0.928(0.006) | 5.16 | 0.000 | 1.149 | 0.697* | 0.662,0.732 |
|  | ≤0.90 | 627 | 0.872(0.006) |  |  |  |  |  |
| EQ-5D-UK | ＞0.90 | 302 | 0.946(0.10) | 4.23 | 0.002 | 0.942 | 0.639* | 0.603,0.675 |
|  | ≤0.90 | 627 | 0.881(0.009) |  |  |  |  |  |
| EQ-5D-JP | ＞0.90 | 302 | 0.945(0.009) | 4.92 | 0.001 | 1.096 | 0.640* | 0.604,0.675 |
|  | ≤0.90 | 627 | 0.882(0.009) |  |  |  |  |  |
| EQ-5D-USA | ＞0.90 | 302 | 0.955(0.008) | 4.49 | 0.001 | 1 | 0.639* | 0.603,0.675 |
|  | ≤0.90 | 627 | 0.906(0.006) |  |  |  |  |  |

* AUC statistically significantly less than 0.5. †Reference is EQ-5D-USA measure. AUC, area under ROC curves; CI, confidence interval; EQ-5D, EuroQol; EQ-VAS, EuroQol Visual Analog Scale; RE, relative efficiency; ROC, receiver operating characteristic; SF-6D, Short Form 6D. EQ-5D-UK, EQ-5D in UK weights; EQ-5D-JP, EQ-5D in Japan weights; EQ-5D-USA, EQ-5D in USA weights.

Table S1b. Multiple linear regression analyses for utility difference between EQ-5Ds and SF-6D

| Difference |  | Non-standardization | | | Standardization | | |
| --- | --- | --- | --- | --- | --- | --- | --- |
| (vs) | Variable | Coefficient (SE) | P value | R^2^ | Coefficient (SE) | P value | R^2^ |
| SF-6D vs | Unmarried^a^ | 0.064(0.026) | 0.033 | 0.035 | 0.011(0.038) | 0.766 | 0.034 |
| EQ-5D-UK | Middle education^b^ | 0.040(0.014) | 0.015 |  | 0.021(0.008) | 0.002 |  |
|  | AMC | -0.033(0.028) | 0.259 |  | -0.067(0.030) | 0.049 |  |
| SF-6D vs | age | 0.001(0.000) | 0.086 | 0.024 | 0.001(0.000) | 0.016 | 0.024 |
| EQ-5D-JP | Unmarried^a^ | 0.059(0.022) | 0.025 |  | 0.034(0.034) | 0.341 |  |
|  | Middle education^b^ | 0.024(0.010) | 0.040 |  | 0.019(0.010) | 0.091 |  |
| SF-6D vs | Middle education^b^ | 0.028(0.009) | 0.014 | 0.031 | 0.022(0.009) | 0.026 | 0.031 |
| EQ-5D-USA | CMC | -0.004(0.010) | 0.675 |  | -0.028(0.007) | 0.004 |  |
| SF-6D vs VAS | age | 0.002(0.001) | 0.024 | 0.032 | 0.002(0.001) | 0.024 | 0.021 |
| EQ-5D-UK vs | Full coverage^c^ | -0.080(0.025) | 0.011 | 0.045 | -0.043(0.009) | 0.001 | 0.040 |
| EQ-5D-JP | Partial coverage^c^ | -0.014(0.004) | 0.007 |  | -0.008(0.006) | 0.182 |  |
|  | AMC | 0.018(0.005) | 0.004 |  | 0.022(0.008) | 0.020 |  |
| EQ-5D-UK vs | Unmarried^a^ | -0.019(0.006) | 0.014 | 0.036 | -0.010(0.002) | 0.001 | 0.027 |
| EQ-5D-USA | Middle education^b^ | -0.012(0.005) | 0.039 |  | 0.001(0.001) | 0.352 |  |
| EQ-5D-JP vs | Full coverage^c^ | 0.038(0.009) | 0.003 | 0.043 | 0.034(0.009) | 0.003 | 0.043 |
| EQ-5D-USA | AMC | -0.019(0.008) | 0.032 |  | -0.017(0.007) | 0.032 |  |
| EQ-5D-UKvs | Partial coverage^c^ | -0.047(0.020) | 0.044 | 0.025 | -0.040(0.016) | 0.030 | 0.035 |
| VAS | AMC | 0.048(0.018) | 0.022 |  | 0.056(0.023) | 0.034 |  |
| EQ-5D-USA | Partial coverage^c^ | -0.041(0.018) | 0.049 | 0.028 | -0.040(0.017) | 0.044 | 0.031 |
| vs VAS | AMC | 0.050(0.021) | 0.038 |  | 0.052(0.022) | 0.041 |  |

^a^divorce as a reference; ^b^higher education as a reference; ^c^no insurance or self-expense as a reference; SE, standard error; EQ-5D, EuroQol; EQ-VAS, EuroQol Visual Analog Scale; SF-6D, Short Form 6D. The difference between scores of utility instruments was dependent variable. Sex, age, marriage, district, family size, education level, income, health insurance coverage, chronic medical condition(CMC), acute medical condition(AMC) as independent variable. The results without statistical significance in the non-standardization scores and constant were not shown. EQ-5D-UK, EQ-5D in UK weights; EQ-5D-JP, EQ-5D in Japan weights; EQ-5D-USA, EQ-5D in USA weights.
